# Spectral-Domain Measurements of Birefringence and Sensing Characteristics of a Side-Hole Microstructured Fiber

**DOI:** 10.3390/s130911424

**Published:** 2013-08-28

**Authors:** Petr Hlubina, Tadeusz Martynkien, Jacek Olszewski, Pawel Mergo, Mariusz Makara, Krzysztof Poturaj, Waclaw Urbańczyk

**Affiliations:** 1 Department of Physics, Technical University Ostrava, 17. listopadu 15, Ostrava-Poruba 708 33, Czech Republic; 2 Institute of Physics, Wroclaw University of Technology, Wybrzeże Wyspiańskiego 27, Wroclaw 50-370, Poland; E-Mails: tadeusz.martynkien@pwr.wroc.pl (T.M.); jacek.olszewski@pwr.wroc.pl (J.O.); waclaw.urbanczyk@pwr.wroc.pl (W.U.); 3 Laboratory of Optical Fibre Technology, Maria Curie-Sklodowska University, Pl. M. Curie-Sklodowskiej 3, Lublin 20-031, Poland; E-Mails: pawel.mergo@poczta.umcs.lublin.pl (P.M.); marmak@hermes.umcs.lublin.pl (M.M.); potkris@hermes.umcs.lublin.pl (K.P.)

**Keywords:** microstructured fibers, birefringent fibers, fiber characterization, spectral interferometry, birefringence dispersion, fiber-optic sensors, polarimetric sensitivity

## Abstract

We experimentally characterized a birefringent side-hole microstructured fiber in the visible wavelength region. The spectral dependence of the group and phase modal birefringence was measured using the methods of spectral interferometry. The phase modal birefringence of the investigated fiber increases with wavelength, but its positive sign is opposite to the sign of the group modal birefringence. We also measured the sensing characteristics of the fiber using a method of tandem spectral interferometry. Spectral interferograms corresponding to different values of a physical parameter were processed to retrieve the spectral phase functions and to determine the spectral dependence of polarimetric sensitivity to strain, temperature and hydrostatic pressure. A negative sign of the polarimetric sensitivity was deduced from the simulation results utilizing the known modal birefringence dispersion of the fiber. Our experimental results show that the investigated fiber has a very high polarimetric sensitivity to hydrostatic pressure, reaching −200 rad × MPa^−1^× m^−1^ at 750 nm.

## 1. Introduction

Highly birefringent (HB) fibers, such as conventional elliptical-core, bow-tie or side-hole fibers, can be used as active elements of fiber-optic sensor configurations utilizing the interference of polarization modes. A physical quantity acting on HB fiber causes a change in the difference of propagation constants of the polarization modes or, equivalently, a change in the phase modal birefringence and, consequently, a change in the phase shift between the polarization modes at the fiber output. The phase shift can be measured by interferometric or polarimetric methods that can also be used in interferometric and polarimetric sensors for measuring different physical quantities, such as temperature, hydrostatic pressure and elongation [1,2]. As an example, conventional side-hole fibers are of interest for pressure sensing [3–5], because of a very high polarimetric sensitivity, which is due to two large air holes in the cladding adjacent to the core region. The holes break the mechanical symmetry of the fiber and are responsible for a high change of the phase modal birefringence when a symmetrical load induced by hydrostatic pressure applied to the fiber cladding is transferred into nonsymmetrical stress distribution in the core region.

Conventional HB fibers exhibit temperature-sensitive birefringence, so that when they are used for sensing other parameters than temperature, such as hydrostatic pressure, the temperature cross-sensitivity affects the measurement accuracy significantly. To overcome this limitation, HB microstructured (MS) fibers with much higher flexibility in shaping the phase modal birefringence and significantly less temperature dependence than conventional HB fibers have emerged as active elements of fiber-optic sensors. The birefringence in MS fibers originates either from breaking the hexagonal symmetry of the fiber structure [6,7] or from internal stress, causing an anisotropy of the refractive index in the fiber core [8]. HB MS fibers made of glass with a uniform composition in the entire cross-section have no thermal stress induced by the difference in thermal expansion coefficients between the doped fiber core and the fiber cladding. Consequently, the polarimetric sensitivity to temperature, which strongly depends on the geometry of the HB MS fiber, can be up to three orders of magnitude lower than in conventional elliptical-core fibers [9,10].

HB MS fibers with very low temperature cross-sensitivity are suitable for hydrostatic pressure sensing [11–14]. As an example, the effect of hydrostatic pressure on the phase modal birefringence has been analyzed for a commercially available HB MS fiber with two large air holes adjacent to the fiber core [15]. It has been shown that the polarimetric sensitivity to hydrostatic pressure is mostly related to pressure-induced stress birefringence in the core region or, equivalently, to the mechanical asymmetry of the microstructured region. In a commercially available HB MS fiber with two large air holes adjacent to the core region, the polarimetric sensitivity to pressure reaches a value of −14 rad × MPa^−1^× m^−1^ at 780 nm [16].

In this paper, we present the results of the measurement of birefringence and the sensing characteristics of a side-hole MS fiber in the visible wavelength region (500–770 nm). First, the spectral dependence of the group and phase modal birefringence is measured using methods of spectral interferometry. Second, the sensing characteristics of the fiber are measured using a method of tandem spectral interferometry. We extend the use of a method [17] based on processing the spectral interferograms, including the equalization wavelength at which spectral interference fringes have the largest period, due to the zero overall group birefringence. From the spectral interferograms, the phase functions corresponding to different values of a physical parameter are retrieved, and the spectral dependence of polarimetric sensitivity to strain, temperature and hydrostatic pressure is determined. A negative sign of the polarimetric sensitivity is deduced from the simulation results, and the polarimetric sensitivity to strain reaches a value of −16 rad × m*ϵ*^−1^× m^−1^ at 750 nm. Similarly, the polarimetric sensitivity to temperature reaches a value of −0.25 rad × K^−1^× m^−1^ at 750 nm. Moreover, the investigated fiber has a very high polarimetric sensitivity to hydrostatic pressure, reaching −200rad × MPa^−1^× m^−1^ at 750 nm.

## 2. Experimental Methods

### 2.1. Measurement of the Modal Birefringence Dispersion

HB fibers are characterized by the phase and group modal birefringence. The phase modal birefringence, *B*(*λ*), is defined as:

(1) $$B(\lambda )=n_{x}\;(\lambda )-n_{y}\;(\lambda )$$

where *n_x_*(*λ*) and *n_y_*(*λ*) are the wavelength-dependent phase effective indices of *x* and *y* polarization modes supported by the HB fiber. Similarly, the group modal birefringence, *G*(*λ*), is defined as:

(2) $$G(\lambda )=N_{x}\;(\lambda )-N_{y}\;(\lambda )=-\lambda^{2}\frac{\text{d}[B(\lambda )/\lambda ]}{\text{d}\lambda }$$

where *N_x_*(*λ*) and *N_y_*(*λ*) are the group effective indices of the polarization modes.

**Figure 1.**
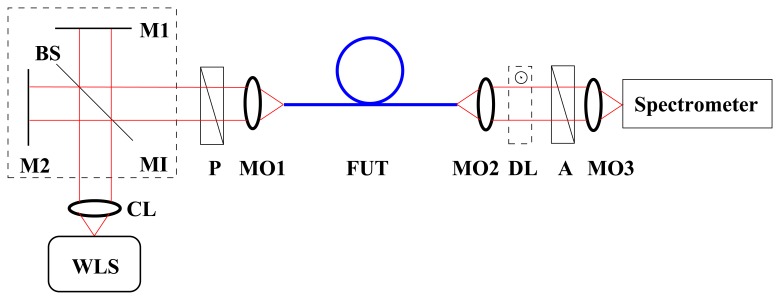
Experimental setup with a Michelson interferometer (MI) for measuring the dispersion of the group modal birefringence in a fiber under test (FUT). The remaining components: a white-light source (WLS), a collimating lens (CL), mirrors 1–2 (M1–2), a beam splitter (BS), a polarizer (P), microscope objectives 1–3 (MO1–3), a delay line (DL) and an analyzer (A).

The group modal birefringence dispersion can be measured by a method of spectral-domain tandem interferometry [18,19] utilizing the experimental setup shown in Figure 1. In this setup, the path length difference, *Δ*_M_, adjusted in a Michelson interferometer (MI) compensates for the group optical path difference, *G*(*λ*)*z*, between the polarization modes in a fiber under test (FUT) of length *z*. The transmission azimuth of both a polarizer and an analyzer is adjusted at 45^°^ with respect to the polarization axes of the FUT, so that the interference of the polarization modes is observed. If we consider *G*(*λ*) < 0 for the FUT, the spectral intensity at the output of the tandem configuration can be expressed as [18,19]:

(3) $$I(z;\lambda )=I_{0}\;(\lambda )\{1+V\;(z;\lambda )\cos\;\{(2\pi /\lambda )[\Delta_{\text{M}}+B\;(\lambda )z]\}\}$$

where *I*_0_(*λ*) is the reference spectral intensity and *V* (*z*; *λ*) is the visibility term given by:

(4) $$V(z;\lambda )=\exp\{-(\pi^{2}/2)\;{\left\{[\Delta_{\text{M}}+G\;(\lambda )z]\Delta \lambda_{\text{R}}{/\lambda }^{2}\right\}}^{2}\}$$

It results from Equation (3) that the spectral interference fringes can be resolved, due to the highest visibility in the vicinity of the equalization wavelength, *λ*_0_ [18], given by:

(5) $$\Delta_{\text{M}}=-G\;(\lambda_{0})\;z$$

Thus, *∆*_M_ adjusted in the MI and measured as a function of the equalization wavelength, *λ*_0_, gives the wavelength dependence of the group modal birefringence, *G*(*λ*_0_) = *∆*_M_/*z*, in the FUT directly. Because the group modal birefringence, *G*(*λ*), is related to the phase modal birefringence, *B*(*λ*), via Equation (2), we can obtain the relative wavelength dependence of the phase modal birefringence. It can be combined with the known value at one specific wavelength to obtain the wavelength dependence of the phase modal birefringence, *B*(*λ*) [18]. The benefits of our spectral methods, compared to the standard frequency-domain interferometer [20], which measures the periodicity of the interference fringes, include no need to use a source of short broadband optical pulses.

### 2.2. Measurement of Polarimetric Sensitivity to Strain, Temperature and Hydrostatic Pressure

**Figure 2.**
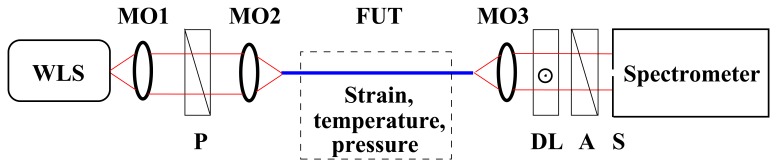
Experimental setup with a fiber under test (FUT) to measure the polarimetric sensitivity to strain, temperature and hydrostatic pressure. The remaining components: a white-light source (WLS), microscope objectives 1–3 (MO1–3), a polarizer (P), a delay line (DL), an analyzer (A) and a slit (S).

To sense a physical parameter (strain, temperature or hydrostatic pressure) acting on the FUT, the configuration of the FUT in tandem with a birefringent crystal of the group birefringence, *G*_c_(*λ*), can be used [21], as shown in Figure 2. The spectral intensity at the output of the tandem configuration with a polarizer and an analyzer oriented 45° with respect to the eigenaxes of the FUT is given for *G*(*λ*) < 0 and *G*_c_(*λ*) > 0 by [21]:

(6) $$I(z;\lambda )=I_{0}(\lambda )\;\{1+V(z;\lambda )\;\cos\;\{(2\pi /\lambda )[B_{c}(\lambda )d+B(\lambda )z]\}\}$$

where *I*_0_(*λ*) is the reference spectral intensity, *B_c_*(*λ*) is the phase birefringence of the crystal and *V*(*z*; *λ*) is the visibility term given by:

(7) $$V(z;\lambda )=\exp\{-(\pi^{2}/2)\;{\left\{[G_{c}(\lambda )d+G(\lambda )z]\Delta \lambda_{\text{R}}/\lambda^{2}\right\}}^{2}\}$$

It results from Equation (7) that the spectral interference fringes can be resolved, due to the highest visibility in the vicinity of the equalization wavelength, *λ*_0_, given by:

(8) $$G_{c}(\lambda_{0})d=-G(\lambda_{0})z$$

From the spectra recorded to sense a specific physical parameter, *X*, the phase shift, ∆[*ϕ_x_*(*λ*) − *ϕ_y_*(*λ*)], between the polarization modes can be retrieved. The polarimetric sensitivity, *K_X_*(*λ*), of the FUT to the physical quantity, *X*, is defined by the following relation:

(9) $$K_{X}(\lambda )=\frac{1}{L}\frac{\text{d}[\phi_{x}(\lambda )-\phi_{y}(\lambda )]}{\text{d}X}$$

and represents an increase in the phase shift between the polarization modes induced by the unit change of the physical quantity, *X*, acting on unit fiber length [16].

## 3. Experimental Configurations

The experimental setup used to measure the group modal birefringence dispersion is shown in Figure 1. It consists of a white-light source (WLS): a halogen lamp, collimating lens (C), a bulk-optic Michelson interferometer with a beam splitter and mirrors 1 and 2, polarizer (P) (LPVIS050, Thorlabs), microscope objectives MO1–3 (10×, 0.30 NA, Meopta), fiber under test (FUT), delay line (DL) represented by a birefringent quartz crystal of a suitable thickness, analyzer (A) (LPVIS050, Thorlabs), a spectrometer (S2000, Ocean Optics) and a personal computer. The polarizer and analyzer are oriented 45° with respect to the fiber eigenaxes.

The experimental setup used to measure the spectral dependence of the polarimetric sensitivity to strain, temperature and hydrostatic pressure is shown in Figure 2. It consists of a white-light source (WLS): a supercontinuum source (NKT Photonics), polarizer (P) (LPVIS050, Thorlabs), fiber under test (FUT), microscope objectives MO1–3 (10×, 0.30 NA), delay line (DL), analyzer (A) (LPVIS050, Thorlabs), a spectrometer (USB4000, Ocean Optics) with a 25 μm-wide slit (S) and a personal computer. To record the spectral intensity, *I*(*z*; *λ*), the polarizer and analyzer are oriented 45° with respect to the fiber eigenaxes, and the optical axis of the birefringent quartz crystal is oriented 0° with respect to the major fiber eigenaxis. Even if many components employed in the experimental setup are bulky and good alignment is necessary, the stability of the measurements is satisfactory.

**Figure 3.**
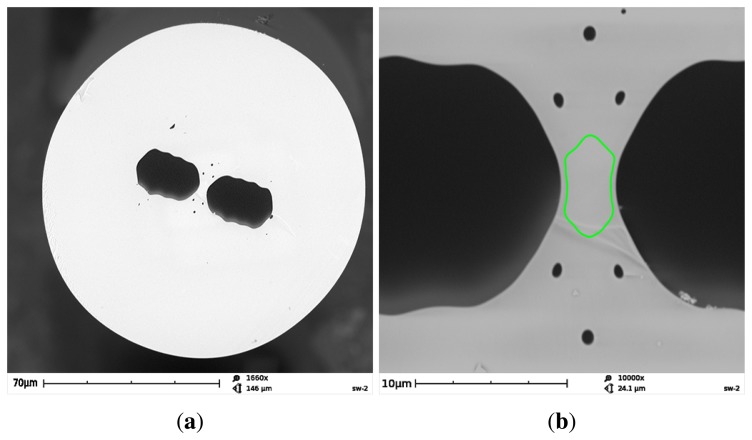
Structure of the FUT: general view of the fiber cross-section (**a**). Enlarged image of the central region with the green line showing the core boundary (**b**).

The FUT is a birefringent side-hole fiber with additional microstructure near the core region composed of six small holes of a diameter of about 0.7 μm, which prevent the guided mode from leaking out from the core. The fiber was drawn at the Department of Optical Fibers Technology, University of Marie Curie-Sklodowska in Lublin, Poland. As is shown in Figure 3, the fiber core has an elliptical shape of the dimensions 6.5 μm × 2.8 μm, while the cladding diameter is 129 μm. The longer axis of the core ellipse is perpendicular to the symmetry axis connecting the centers of the large holes. The initial GeO_2_ concentration in the rod used to preform fabrication was 12 mol%, which corresponds to the refractive index contrast of 0.017 at 633 nm. However, due to material flow and GeO_2_ diffusion during the fiber drawing process, it is expected that final GeO_2_ concentration in the doped region of the fiber core is much lower. As the glass bridges between the large holes and the doped region of the core are very narrow (about 0.4–0.5 μm), this results in the cutoff of the fundamental mode at about 800 nm.

## 4. Experimental Results and Discussion

### 4.1. Phase and Group Modal Birefringence Dispersion

**Figure 4.**
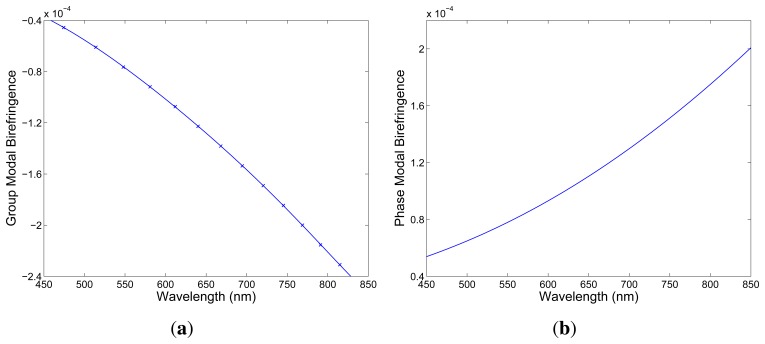
Measured spectral dependence of the group (**a**) and phase (**b**) modal birefringence in the FUT. The line crossing the markers is a polynomial fit.

The results of the measurement of the group modal birefringence dispersion, *G*(*λ*), in the experimental setup shown in Figure 1 are presented in Figure 4a by markers. The negative sign of the group modal birefringence is specified using a simple procedure with a delay line (DL) [19]. The precision in obtaining the group modal birefringence is better than 0.1% [19]. In the same figure is also shown a polynomial fit used to obtain the relative spectral dependence of the phase modal birefringence. It was combined with the known value, *B* = 1.16 × 10^−4^, measured at *λ* = 665 nm by a lateral force method applied in the spectral domain [22] to obtain the spectral dependence of the phase modal birefringence, *B*(*λ*), which is shown in Figure 4b. The phase modal birefringence increases with wavelength, but its sign is opposite to the sign of the group modal birefringence, as is standard for HB MS fibers [19].

### 4.2. Polarimetric Sensitivity to Strain, Temperature and Hydrostatic Pressure

**Figure 5.**
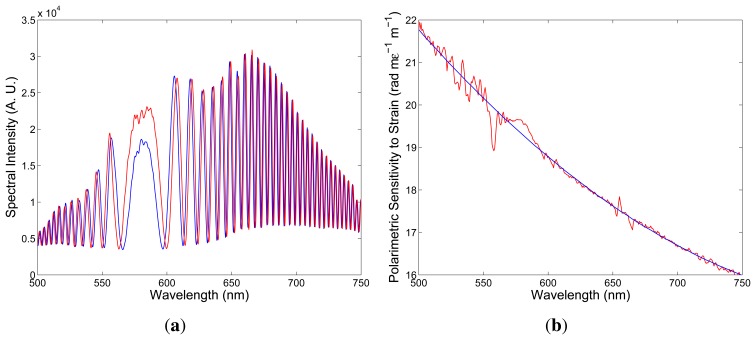
Examples of the recorded spectra corresponding to two elongations, ∆*L*_1_= 100 μm (blue) and ∆*L*_2_ = 150 μm (red), of the FUT (**a**). The spectral dependence of the absolute value of the polarimetric sensitivity to strain (**b**). The line is the approximate quadratic dependence.

To determine the polarimetric sensitivity to strain, *K_ϵ_*(*λ*), we recorded a sequence of spectral interferograms for increasing strain *ϵ* with a step small enough to assure unambiguity in retrieving the strain-induced phase changes, ∆[*ϕ_x_*(*λ*) − *ϕ_y_*(*λ*)]. To measure the parameter, a fiber of length *L* = 0.895 m was attached with epoxy glue to two translation stages and elongated up to 800 μm (stretched up to 894 μ*ϵ*). The measurements were repeated several times for increasing and decreasing strain, with no hysteresis observed. Figure 5a shows two recorded spectra obtained for a suitably chosen delay line and corresponding to two elongations ∆*L*_1_= 100 μm and ∆*L*_2_ = 150 μm, of the fiber when the overall length of the fiber, including also the sensing part, with length *L* = 0.895 m, was *z* = 2.295 m. It is clearly seen from the figure that the interference of polarization modes in tandem with the delay line shows up as the spectral modulation with the wavelength-dependent period and the equalization wavelength, *λ*_0_ = 581.61 nm. The spectral interference fringes for the two elongations are with the same equalization wavelength, but with different phases.

Using a new procedure [23], we retrieved from the two spectral interferograms the phase functions that are wavelength-dependent, with a minimum at the equalization wavelength, *λ*_0_. From the phase functions retrieved from several recorded spectral interferograms, the spectral dependence of the polarimetric sensitivity to strain *K_ϵ_* was obtained, as shown in Figure 5b by the red curve. The polynomial approximation of the absolute mean value of the polarimetric sensitivity as a function of wavelength is shown in the same figure by the blue line. The relative error of the measured polarimetric sensitivity to strain is about 5%.

**Figure 6.**
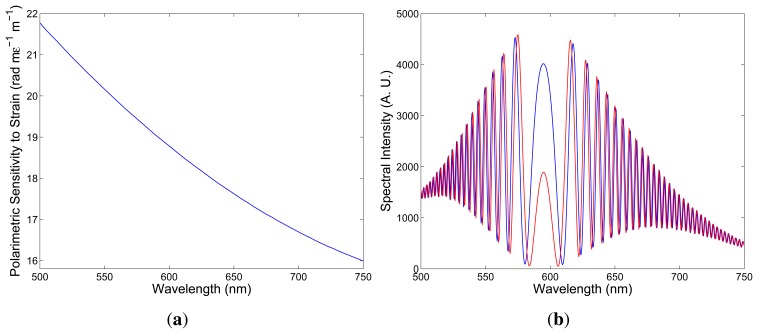
The spectral dependence of the polarimetric sensitivity to strain: a positive sign (**a**). Two theoretical spectra corresponding to the fiber elongations, ∆*L* = 100 μm (blue) and ∆*L* = 150 μm (red) (**b**).

The sign of the polarimetric sensitivity can be deduced from the results of the subsequent simulation. First, consider a positive sign of the polarimetric sensitivity to strain, as shown in Figure 6a and simulate the spectral interferogram, *I*(*z*; *λ*), according to Equation (6). First, we consider the fiber elongation, ∆*L* = 100 μm, so that the strain-induced phase change is:

(10) $$\Delta [\phi_{x}(\lambda )-\phi_{y}(\lambda )]=K_{\epsilon }(\lambda )\Delta L$$

Using the well known birefringence dispersion for the quartz crystal [21] and the measured birefringence dispersion for the FUT (see Figures 4), we obtained for the fiber length, *z* = 2.35 m, and for the crystal thickness, *d* = 23.5 mm, the spectral interferogram, *I*(*z*; *λ*), shown in Figure 6b by the blue curve. Next, we consider the fiber elongation, ∆*L* = 150 μm, and the corresponding spectral interferogram, *I*(*z*; *λ*), is shown in Figure 6b by the red curve. It is clearly seen that the spectral interference fringes are shifting to the equalization wavelength with the increasing elongation.

**Figure 7.**
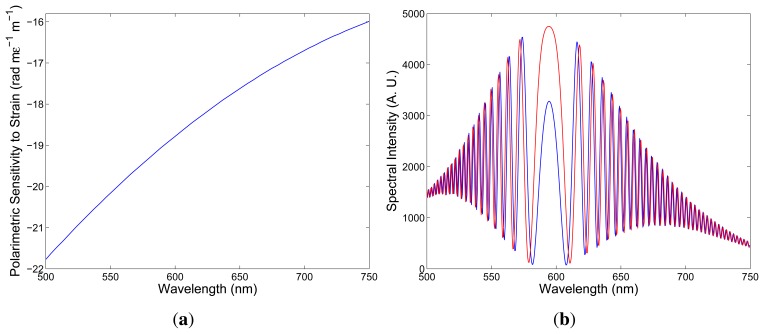
The spectral dependence of the polarimetric sensitivity to strain: a negative sign (**a**). Two theoretical spectra corresponding to the fiber elongations, ∆*L* = 100 μm (blue) and ∆*L* = 150 μm (red) (**b**).

Second, consider a negative sign of the polarimetric sensitivity to strain, as shown in Figure 7a, and simulate the change of the spectral interferogram, *I*(*z*; *λ*), with elongation. Using the same parameters – the fiber length, *z* = 2.35 m, and the crystal thickness, *d* = 23.5 mm – we obtained for the fiber elongation, ∆*L* = 100 μm, the spectral interferogram, *I*(*z*; *λ*), shown in Figure 7b by the blue curve. Next, we consider the fiber elongation, ∆*L* = 150 μm, and Figure 7b shows the corresponding spectral interferogram, *I*(*z*; *λ*), by the red curve. In this case, the spectral interference fringes are shifting from the equalization wavelength with the increasing elongation.

Comparing the spectral interferograms recorded for the increasing elongation (see Figure 5a) to the simulated spectral interferograms corresponding to different signs of the polarimetric sensitivity to strain (see Figures 6b and 7b), we conclude that the polarimetric sensitivity to strain is negative, and its absolute value decreases with wavelength, from a value of 21.8 rad × m*ϵ*^−1^ × m^−1^ to a value of 16.0 rad x m*ϵ*^−1^ × m^−1^ (in a range from 500 to 750 nm). A negative sign of the polarimetric sensitivity to strain indicates a decrease of the phase modal birefringence with strain. The polarimetric sensitivity of the investigated fiber to strain is comparable to that of conventional elliptical-core HBs [24].

**Figure 8.**
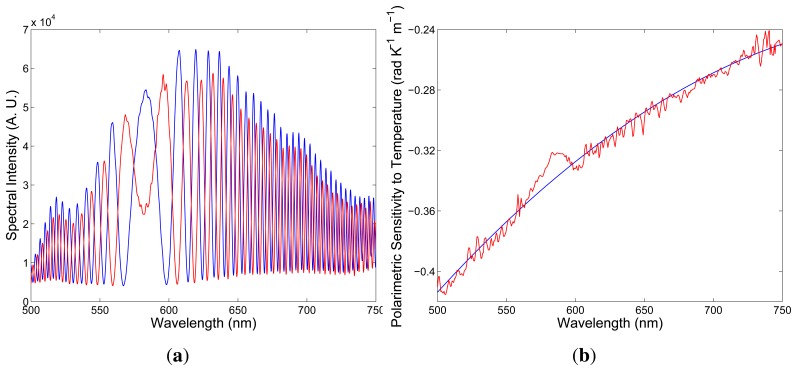
Two recorded spectra corresponding to temperatures *T*_1_ = 297 **K** (blue) and *T*_2_ = 333 **K** (red) (**a**). The spectral dependence of the polarimetric sensitivity to temperature (**b**). The line is the approximate quadratic dependence.

To determine the polarimetric sensitivity to temperature, *K_T_*(*λ*), we recorded a sequence of spectral interferograms for increasing temperature, *T*, with a step small enough to assure unambiguity in retrieving the temperature-induced phase changes, ∆[*ϕ_x_*(*λ*) − *ϕ_y_*(*λ*)]. To measure *K_T_*(*λ*), a bare fiber of length *L* = 0.225 m was immersed in water heated in a chamber with increasing temperature up to 373 K. Figure 8a shows two recorded spectra obtained for a suitably chosen thickness of the birefringent crystal and corresponding to temperatures *T*_1_ = 297 K and *T*_2_ = 333 K, when the overall length of the fiber was *z* = 1.620 m. It is clearly seen from the figure that the interference of polarization modes in tandem with the birefringent crystal shows up as the spectral modulation with the wavelength-dependent period and the equalization wavelength, *λ*_0_ = 583.02 nm. The spectral interference fringes for the two temperatures are with the same equalization wavelength, but with different phases.

We retrieved from the two spectral interferograms the phase functions that are wavelength-dependent with a minimum at the equalization wavelength, *λ*_0_. From the phase functions retrieved from several recorded spectral interferograms, the spectral dependence of the polarimetric sensitivity to temperature, *K_T_*(*λ*), was obtained, as shown in Figure 8b by the red curve. The polynomial approximation of the mean value of the polarimetric sensitivity as a function of wavelength is shown in the same figure by the blue line. The relative error of the measured polarimetric sensitivity to temperature is about 5%. The negative sign of the polarimetric sensitivity can be once again deduced from the results of the simulation. Similarly, as for the strain, the spectral interference fringes are shifting from the equalization wavelength with increasing temperature. A negative sign of the polarimetric sensitivity to temperature indicates a decrease of the phase modal birefringence with temperature, due to the release of thermal stress in the core region. The polarimetric sensitivity of the investigated fiber to temperature is comparable to that of conventional elliptical-core HBs [25].

**Figure 9.**
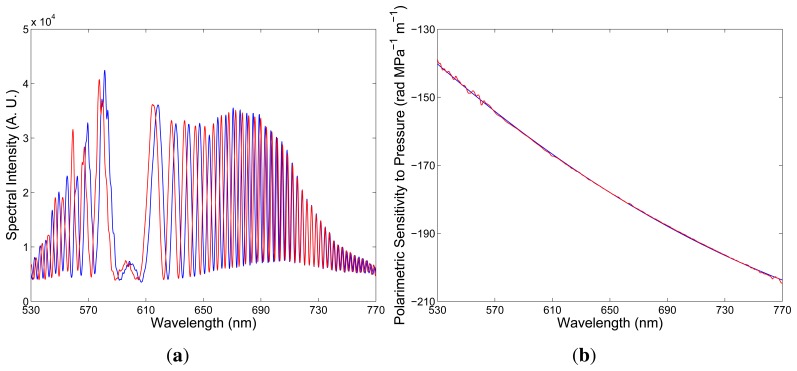
Two recorded spectra corresponding to the first delay and hydrostatic pressures, *p*_1_ = 0.72 MPa (blue) and *p*_2_ = 1.05 MPa (red) (**a**). The spectral dependence of the polarimetric sensitivity to hydrostatic pressure (**b**). The red line is the approximate quadratic dependence.

To determine the polarimetric sensitivity to hydrostatic pressure, *K_p_*(*λ*), we used a rather different procedure. We recorded a sequence of spectral interferograms for increasing pressure, *p*, with the known pressure-induced phase changes, ∆[*ϕ_x_*(*λ*_0_) − *ϕ_y_*(*λ*_0_)], e.g., 10*π* at the equalization wavelength, *λ*_0_. The sensing length of the fiber in a pressure chamber was *L* = 0.648 m, and measurement of the polarimetric sensitivity to hydrostatic pressure was performed up to 2.3 MPa. Figure 9a shows two recorded spectra obtained for a suitably chosen thickness of the birefringent crystal and corresponding to hydrostatic pressures *p*_1_ = 0.72 MPa and *p*_2_ = 1.05 MPa when the overall length of the fiber was *z* = 1.129 m. It is clearly seen from the figure that for the hydrostatic pressure, *p*_1_= 0.72 MPa, the interference of polarization modes in tandem with the birefringent crystal shows up as the spectral modulation with the wavelength-dependent period and the equalization wavelength, *λ*_0_ = 597.05 nm. For the hydrostatic pressure, *p*_1_ = 1.05 MPa, the equalization wavelength is changed, due to the group modal birefringence change [26].

We retrieved from the spectral interferogram the phase function, and the same procedure was used for the spectral interferogram corresponding to the hydrostatic pressure, *p*_2_ = 1.05 MPa, when the known phase change was 10*π* at the equalization wavelength, *λ*_0_. From the phase functions retrieved from several spectral interferograms recorded for the known phase change at the equalization wavelength, *λ*_0_, the spectral dependence of the polarimetric sensitivity to hydrostatic pressure, *K_p_*(*λ*), was obtained, as shown in Figure 9b by the red curve. The quadratic approximation (blue line) of the mean value of the spectral dependence of the polarimetric sensitivity to hydrostatic pressure is shown in Figure 9b, and the relative error is about 1%. A negative sign of the polarimetric sensitivity to hydrostatic pressure can be deduced from the simulation results. The sign indicates that pressure-induced material birefringence in the core region has an opposite sign to the geometrical birefringence, due to core ellipticity. The absolute value of the polarimetric sensitivity to hydrostatic pressure increases with wavelength from a value of 140.6 rad × MPa^−1^ × m^−1^ to a value of 203.7 rad × MPa^−1^ × m^−1^ (in a range from 530 to 770 nm). For comparison, a side-hole MS fiber with the polarimetric sensitivity of about −220 rad × MPa^−1^× m^−1^ at *λ* = 700 nm and −100 rad × MPa^−1^× m^−1^ at *λ* = 1.55 μm was reported [14].

**Figure 10.**
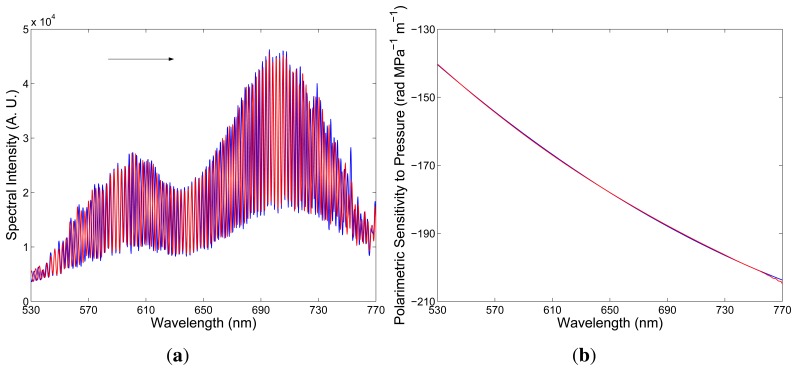
Two recorded spectra corresponding to the second delay and hydrostatic pressures, *p*_1_ = 0.3 MPa (blue) and *p*_2_ = 0.99 MPa (red) (**a**). The spectral dependence of the polarimetric sensitivity to hydrostatic pressure (**b**). The red line is the approximate quadratic dependence.

We also measured the polarimetric sensitivity to hydrostatic pressure, *K_p_*(*λ*), with a different thickness of the birefringent crystal when no equalization wavelength is resolvable in the recorded spectrum and when the pressure-induced phase change is known at a specific wavelength. In this case, the spectral fringes of slightly wavelength-dependent period are resolved in the recorded spectrum, and the spectral polarimetric sensitivity to hydrostatic pressure, *K_p_*(*λ*), is determined from the change of spectral phase with the hydrostatic pressure. As an example, Figure 10b shows two examples of the recorded spectra corresponding to hydrostatic pressures *p*_1_ = 0.3 MPa and *p*_2_ = 0.99 MPa, when the specific wavelength is 641.93 nm and the spectral fringes are shifted with the increasing hydrostatic pressure to longer wavelengths. It is clearly seen from the figure that the interference of polarization modes in tandem with the delay line shows up as the spectral modulation with no equalization wavelength in the considered spectral region. The spectral interference fringes for the two pressures are with different phases, which were retrieved from the two spectral interferograms using a procedure based on the application of a windowed Fourier transform [27]. Figure 10b shows by the red curve the spectral dependence of the polarimetric sensitivity to hydrostatic pressure, *K_p_*(*λ*), together with the approximate quadratic dependence (blue line). This approach gives the polarimetric sensitivity with errors smaller in comparison with those obtained by the first approach.

## 5. Conclusions

We measured the birefringence and sensing characteristics of a side-hole MS fiber in the visible wavelength region (500–770 nm). The spectral dependence of the group and phase modal birefringence is measured using the methods of spectral interferometry. The sensing characteristics of the fiber are measured using a method of tandem spectral interferometry. Spectral interferograms corresponding to different values of a physical parameter are processed to retrieve the spectral phase functions. These are used to determine the spectral dependence of polarimetric sensitivity to strain, temperature and hydrostatic pressure. A negative sign of the polarimetric sensitivity is deduced from the simulation results utilizing the known modal birefringence dispersion of the fiber.

The polarimetric sensitivity of the investigated fiber to strain reaches *K_ϵ_* = −16 rad × m*ϵ*^−1^× m^−1^ at *λ* = 750 nm, which is comparable to that of conventional HBs with an elliptical core. Similarly, the polarimetric sensitivity to temperature reaches *K_T_* = −0.25 rad × K^−1^× m^−1^ at *λ* = 750 nm, which is comparable to that of conventional HBs with an elliptical core. The investigated fiber has a very high polarimetric sensitivity to hydrostatic pressure, reaching *K_p_* = −200 rad × MPa^−1^× m^−1^ at *λ* = 750 nm. The ratio, *K_p_*/*K_T_*, which is an important figure of merit for the investigated fiber, reaches a value of 800 K× MPa^−1^ at *λ* = 750 nm, so that the fiber is suitable for hydrostatic pressure measurements with low cross-sensitivity to temperature.
